# Hypoxia Strongly Affects Mitochondrial Ribosomal Proteins and Translocases, as Shown by Quantitative Proteomics of HeLa Cells

**DOI:** 10.1155/2015/678527

**Published:** 2015-09-02

**Authors:** Paula A. Bousquet, Joe Alexander Sandvik, Magnus Ø. Arntzen, Nina F. Jeppesen Edin, Stine Christoffersen, Ute Krengel, Erik O. Pettersen, Bernd Thiede

**Affiliations:** ^1^Department of Chemistry, University of Oslo, P.O. Box 1033 Blindern, 0315 Oslo, Norway; ^2^Department of Physics, University of Oslo, P.O. Box 1048 Blindern, 0315 Oslo, Norway; ^3^The Biotechnology Centre of Oslo, University of Oslo, P.O. Box 1125 Blindern, 0317 Oslo, Norway; ^4^Department of Biosciences, University of Oslo, P.O. Box 1066 Blindern, 0316 Oslo, Norway

## Abstract

Hypoxia is an important and common characteristic of many human tumors. It is a challenge clinically due to the correlation with poor prognosis and resistance to radiation and chemotherapy. Understanding the biochemical response to hypoxia would facilitate the development of novel therapeutics for cancer treatment. Here, we investigate alterations in gene expression in response to hypoxia by quantitative proteome analysis using stable isotope labeling with amino acids in cell culture (SILAC) in conjunction with LCMS/MS. Human HeLa cells were kept either in a hypoxic environment or under normoxic conditions. 125 proteins were found to be regulated, with maximum alteration of 18-fold. In particular, three clusters of differentially regulated proteins were identified, showing significant upregulation of glycolysis and downregulation of mitochondrial ribosomal proteins and translocases. This interaction is likely orchestrated by HIF-1. We also investigated the effect of hypoxia on the cell cycle, which shows accumulation in G1 and a prolonged S phase under these conditions. *Implications*. This work not only improves our understanding of the response to hypoxia, but also reveals proteins important for malignant progression, which may be targeted in future therapies.

## 1. Introduction

Hypoxia is defined by low oxygen levels (<5%). It is a common characteristic of most advanced tumors but is also naturally occurring during normal embryogenesis [1, 2]. It has been known for many years that hypoxia has a negative impact on the effectiveness of radiation and chemotherapy [1, 3]. More recently, hypoxia has been recognized as a major factor impacting malignant progression, that is, an increased probability of metastasis and recurrence [4]. Even though hypoxia can affect tumor growth negatively, the cellular responses induced by hypoxia may lead to an enhanced aggressiveness of the tumor, resulting in poor prognosis. This is partly due to elevated mutation frequency, increased genetic instability, and an enhanced metastatic potential [5, 6] and partly due to resistance to radiation and chemotherapy. Hypoxia-induced malignant progression concurs with alterations from the genome to the proteome level, resulting in activating processes that allow the tumor to escape and survive in an environment deficient in oxygen. Transcription factor HIF-1 is a master regulator of adaptation to hypoxia, accountable for regulating metabolic processes including energy metabolism, oxygen homeostasis, growth, and differentiation [7, 8]. It is responsible for shifting the cell energy production by increasing the expression of glycolytic genes. The fact that utilization of glucose is more rapid in the absence of oxygen was first demonstrated by Louis Pasteur in 1861 and is termed the “Pasteur effect” [9]. Almost a century ago, Otto Warburg reported that cancer metabolism is different from that of normal tissues, showing that, in tumor cells, over 50% of the ATP is generated by glycolysis, compared to 10% in normal cells [10]. Aerobic glycolysis is uniquely increased in cancer cells, which led Warburg et al. to the hypothesis that mitochondrial dysfunction could result in cancer [11]. This hypothesis has proven incorrect, but a number of other mechanisms promoting increased glycolysis have been proposed, including the activation of oncogenes, mutations in tumor suppressors, and hypoxic adaptations [12–14].

Hypoxia is an important source of stress for tumor cells, and a number of response and survival pathways are thought to be essential for cancer cells to overcome this stress factor. Several studies have been performed to reveal the transcriptome of tumor cells under hypoxic conditions [15–17]. Proteomic studies to reveal the response to hypoxia in tumor cells have been limited [18–22]. One proteome study analyzed hypoxia-induced changes in mouse 4T1 mammary cancer cells using stable isotope labeling with amino acids in cell culture (SILAC) in conjunction with LCMS/MS [22]. We employed the same large-scale proteomic approach to monitor altered protein ratios in HeLa cells under hypoxic conditions. This technique enables comprehensive protein identification by providing a defined number of labels per peptide [23, 24]. Applying this method, 125 proteins showed statistically significant quantitative changes, 72 of which were upregulated and 53 downregulated. Proteins exhibiting significant alteration are involved in metabolism, transport, and signaling.

## 2. Materials and Methods

### 2.1. Cell Cultivation

HeLa P cells were grown in Dulbecco's modified Eagle's medium (DMEM) high glucose, supplemented with 10% fetal bovine serum (FBS) and 1% penicillin/streptomycin. For SILAC experiments, cells were cultured for at least five cell doublings in media supplemented with 10% dialyzed FBS and either ^13^C_6_^15^N_4_-labeled L-arginine (Arginine-10) and ^13^C_6_-labeled L-lysine (Lysine-6) or media containing unlabeled L-arginine and L-lysine amino acids (Thermo Scientific).

### 2.2. Oxygen Sensing

Cells cultured in media containing unlabeled amino acids (“light”) in 19% O_2_ were used as control and compared with isotopically labeled (“heavy”) cells grown in hypoxia (1% O_2_). Cells were seeded in 75 cm^2^ flasks and were harvested after 72 h. The method used to determine pericellular oxygen concentration and cellular respiration/consumption was described previously [25] and is described in more detail in the Supplementary Information in Supplementary Material available online at http://dx.doi.org/10.1155/2015/678527.

### 2.3. DNA/Cell Cycle Analysis

HeLa cells grown for 72 h in hypoxia or normoxia were trypsinized and thereafter washed twice with a solution containing 49 mL phosphate buffered saline (PBS), 1 mL FBS, and 200 *μ*L EDTA before fixation with 4% paraformaldehyde for 15 minutes. After a third washing step, the cells were incubated in 300 *μ*L of PBS containing 0.1 mg/mL RNAse and 0.014 mg/mL propidium iodide for 15 minutes in the dark. The samples were then filtered and analyzed by flow cytometry (BD Accuri C6 flow cytometer) using the Multicycle AV DNA analysis software (FCS Express).

### 2.4. NanoLC-LTQ Orbitrap Mass Spectrometry

The cell lysates from each labeling, heavy and light, were mixed 1 : 1, subjected to SDS-PAGE, trypsinized, and subjected to NanoLC-LTQ Orbitrap mass spectrometry analysis, as described in detail in the Supplementary Information.

### 2.5. Protein Identification and Quantification

Protein identification and quantification were performed with MaxQuant [26] (v.1.2.2.5) utilizing the Andromeda search engine [27] with the IPI human database (v.3.68-87.061 human sequences) including common contaminants. For estimation of the false discovery rate (FDR), we included the reversed sequences into the database search. All hits to the reversed database could thus be regarded as false hits. By restricting the number of matches to this database to only 1% of total matches, we thus proceeded with an FDR of 1% to ensure reliable protein identification. For quantification, at least two quantification events were required per protein, and we further required the proteins to be quantified in at least 2 of 3 replicates. All normalized protein ratios were subject to *z*-statistics for estimation of ratio significances, and a Benjamini-Hochberg correction for multiple hypothesis testing was applied according to Cox and Mann [26]. Proteins with corrected *p* values < 0.1 were regarded differentially altered to avoid missing important proteins at the border of the commonly used statistical limit of *p* < 0.05. These proteins were subsequently used as input to DAVID [28, 29], where the enrichment score served as a more stringent statistical criterion. Significance was further ensured by identification of protein clusters. Further details are given in the Supplementary Information.

The mass spectrometry proteomics data have been deposited to the ProteomeXchange Consortium [30]* via* the PRIDE partner repository with dataset identifier PXD002001. Selected proteins were subjected to Western blotting (Figure S4), confirming the proteomics results.

### 2.6. Bioinformatics Analysis

Functional annotation was performed using DAVID Bioinformatics Resources version 6.7 [28, 29] available at http://david.abcc.ncifcrf.gov/ using the identified proteins as background. The STRING database (http://string-db.org/) (version 9.1) provides known and predicted protein associations resulting in networks covering > 1100 organisms [31] and was used to visualize protein-protein interactions between the hypoxia-regulated proteins.

## 3. Results and Discussion

Hypoxia has a profound effect on cancer progression and therapy by promoting a more malignant phenotype and causing resistance to standard therapies. In this study we investigated differences in protein expression of HeLa cells under hypoxic conditions with quantitative proteomics and subsequent bioinformatics data analysis. An overview of the experimental strategy is depicted in Figure S1. In total, 3,260 proteins were identified with a false discovery rate (FDR) of 1%, of which 125 were differentially altered with statistical significance (Table S1; see also* Materials and Methods*). Subsequently, functional annotation and classification using DAVID and PANTHER [28, 29, 32] and protein-protein interaction analysis using STRING [31] were carried out for altered proteins.

### 3.1. Pericellular Oxygen Concentration and Cellular Respiration

**Figure 1:**
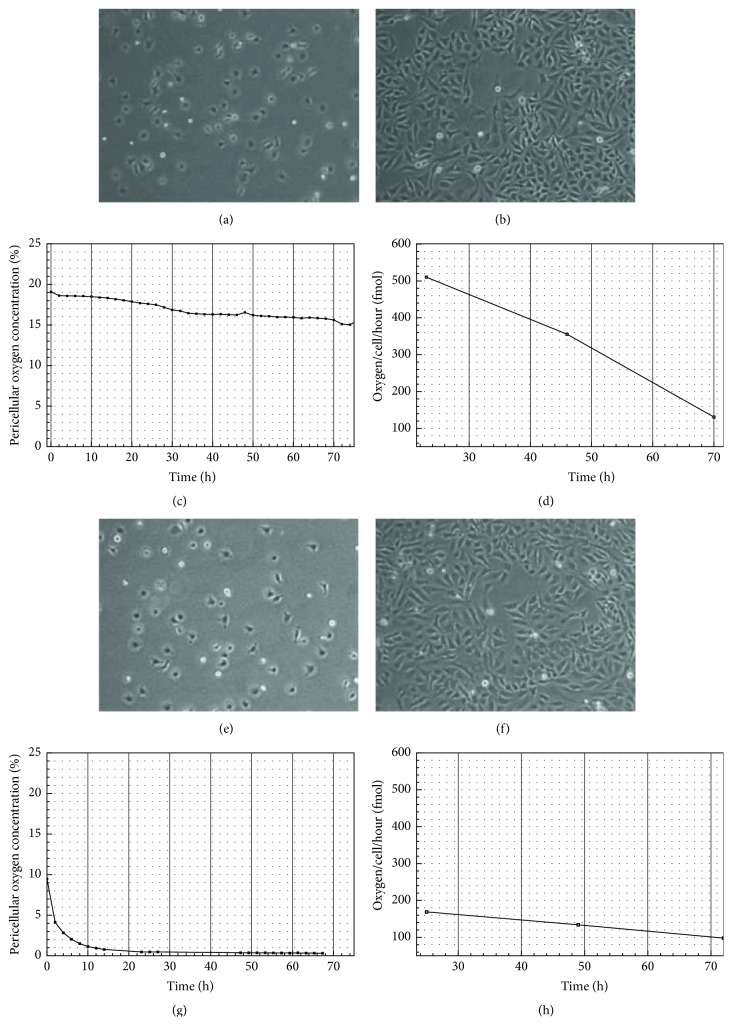
HeLa cells under normoxic (a–d) and hypoxic (e–h) conditions. Panels (a) and (e) represent time point 0, and (b) and (f) show cells after 72 h in normoxic or hypoxic environments, respectively. Panels (c) and (g) show the oxygen profile over a period of 72 h, where the pericellular oxygen concentration was measured with an automated microsensor. The calculated usage of oxygen per cell per hour is depicted in panels 2(d) and (h).

HeLa cells were cultivated in a hypoxia box, where an automated Unisense microsensor was used to measure the oxygen concentration at the bottom of the flask, in close proximity to the cell membrane (Figure 1). Under hypoxic conditions (Figures 1(e)–1(h)), oxygen concentration in the gas phase was 1% throughout the duration of the experiment (72 h); however, the oxygen concentration adjacent to the cell membrane decreased quickly and was much lower during most of the experiment (Figure 1(g)). This is in contrast to normoxic conditions, where the pericellular oxygen concentration decreased only slightly and stabilized at 17 ± 2% (Figure 1(c)), which is close to the concentration of oxygen in the gas phase (19%). Under both hypoxic and normoxic conditions, oxygen usage decreased linearly (Figures 1(d) and 1(h)). Despite the fact that tumor cells in hypoxia show alterations in metabolism, we observed no significant difference in glucose and lactate concentrations under normoxic or hypoxic conditions until the end of the experiment (Figure S2). In both cases, lactate concentrations increased by about 6-fold, whereas glucose concentrations decreased by approximately one-quarter.

### 3.2. Quantitative Proteome Analysis, Functional Annotation, and Classification

To categorize the deregulated proteins identified by SILAC, the up- and downregulated proteins were subjected to functional annotation clustering. As expected, the main biological process affected by hypoxia was metabolism (Figures S3A-B). This is reflected, for example, in the upregulated KEGG pathways, which are all related to metabolic processes (Table S2A). Catalytic activity and binding dominate the molecular functions of both up- and downregulated proteins, with catalytic activity mainly being upregulated and binding activity downregulated (Figures S3C-D). Another large group of proteins that is downregulated relates to structural molecule activity (Figure S3D). Table S2B displays the functional annotation cluster analysis of proteins from HeLa cells under hypoxic conditions. Enrichment scores included in the table are used to rank the biological significance based on the member's *p* values in the corresponding annotation cluster. Glycolysis was, as expected, found to be upregulated. Likewise, oxidoreductase activity and response to hypoxia were identified as upregulated functional annotations, whereas 29 proteins associated with mitochondria were downregulated.

### 3.3. Protein-Protein Interaction Analysis of Changed Proteins

**Figure 2:**
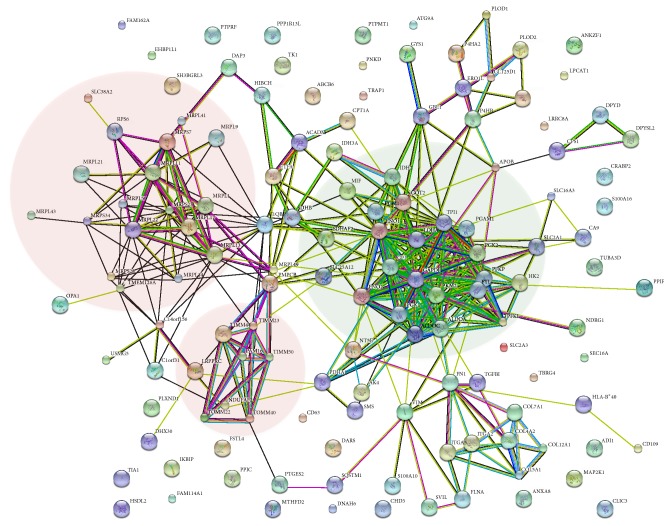
Protein-protein interaction analysis using STRING. Nodes and edges are colored according to type of evidence; protein structures are sketched in the circles. Dark green: neighborhood; red: gene fusion; dark blue: cooccurrence; dark purple: coexpression; light purple: experiments; light blue/green: databases; light green: text-mining; light blue: homology. The gene names are matched to Uniprot accession numbers in Table S1. The backgrounds of up- and downregulated clusters are the shaded backgrounds in red and green, respectively (right: glycolysis, upregulated; bottom: mitochondrial translocases and left: mitochondrial ribosomal proteins, both downregulated).

A network of protein-protein interactions containing the 125 regulated proteins was mapped using STRING (http://string.embl.de/) [31] (Figure 2). A cluster of upregulated proteins involved in glycolysis is connected to two clusters of downregulated mitochondrial ribosomal proteins (MRPs) and translocases of the inner and outer mitochondria membrane (TIMM/TOMMs). To our knowledge, these proteins have not previously been reported as regulated by hypoxia in tumor cells. In the following, different cellular processes affected by hypoxia in HeLa cells were analyzed in more detail.

### 3.4. Upregulation of Anaerobic Glycolysis and Downregulation of Respiration

**Figure 3:**
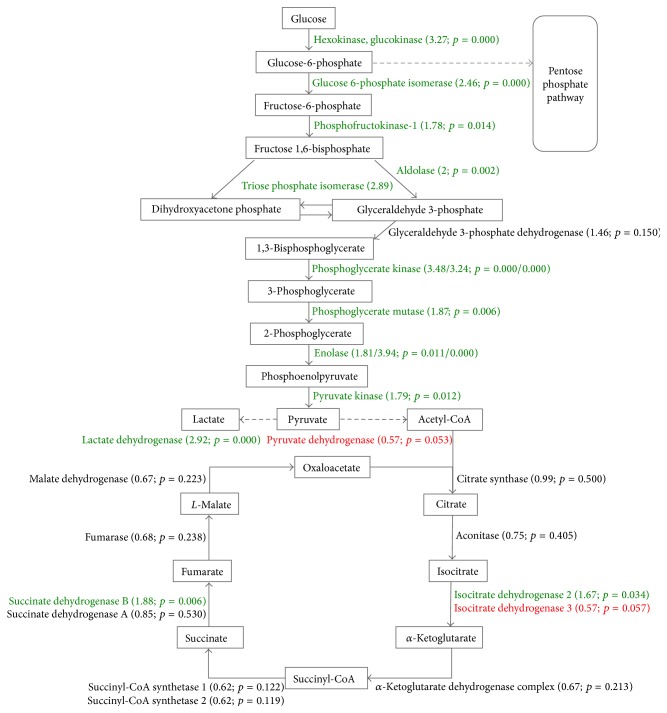
Glycolysis/gluconeogenesis and citric acid cycle. H/L ratios and corrected *p* values are given in parentheses. Proteins that are up- and downregulated with statistical significance are depicted in green and red, respectively, while proteins in black do not satisfy the statistical criteria applied.

Upregulation of glycolysis occurs by upregulation of enzymes involved in the breakdown of glucose or an increase in extracellular glucose import. This was already suggested by earlier genomic [33–35] and proteomic [19, 21, 22, 36] studies and confirmed in the present work, with both glycolytic enzymes and glucose transporters upregulated (Table S1). We found that 72 h of hypoxic exposure increased the protein levels for all proteins of glycolysis (Figure 3). No change was observed for the enzymes specific to gluconeogenesis. The only enzyme found to be downregulated with statistical significance is pyruvate dehydrogenase, which links glycolysis with cellular respiration. This is in keeping with results from Ren et al. [21]. In addition, both of our groups find that several enzymes of the citric acid cycle are downregulated in hypoxic conditions (although satisfying less stringent statistical criteria), with two exceptions: one of the isocitrate dehydrogenases and succinate dehydrogenase (subunit B) exhibit increased levels (with statistical significance). While the citric acid cycle and entry into this pathway were downregulated, lactate dehydrogenase, which converts pyruvate to lactate, the end product of anaerobic glycolysis, was upregulated.

### 3.5. Prevention of Cellular Acidification

Increased glycolysis will result in an accumulation of pyruvate and ultimately lactate, in the cytosol, as observed (Figure S2), which needs to be removed by cotransport with a proton to prevent intracellular acidification. Here we observe that monocarboxylate transporter MCT4/5, which exports lactate from the cell, exhibits increased levels (H/L ratio 1.97). These findings are consistent with a recent article reporting upregulated promoter activity of MCT4 in response to hypoxic stimulation [37]. Another protein preventing acidification of the cell is carbonic anhydrase IX, the protein, which was most strongly upregulated in our study (H/L ratio 17.6 CA IX).

### 3.6. Downregulation of Mitochondrial Proteins

In our study, a total of 29 mitochondrial proteins were found to be downregulated after 72 h of hypoxia (Table S2). An important group of these (16 members) are mitochondrial ribosomal proteins (MRPs). These are components of the mammalian mitochondrial ribosome, which synthesizes in total 13 proteins, all of which are essential subunits of the oxidative phosphorylation complexes [38]. While multiple mitochondrial ribosomal proteins were found to be significantly downregulated, 55 cytosolic ribosomal proteins were identified as nonregulated. In addition, we identified six mitochondrial translocases to be downregulated under hypoxic conditions (Table S1). These proteins are important for the import of nuclear encoded mitochondrial proteins into mitochondria from the cytosol, as only few mitochondrial proteins are encoded by mitochondrial DNA. The responsible transport machinery consists of translocases of the outer and inner mitochondrial membrane (TOMM/TIMM) complexes and has the potential to affect the mitochondrial protein profile [39–41]. The downregulation of MRPL17 and Tim50 was confirmed by Western blotting (Figure S4).

### 3.7. Upregulation of Proteins with Oxidoreductase Activity

Proteins involved in oxidoreductase activity (P4HA1, P4HA2, P4HB, PLOD1, and PLOD2) were found to be upregulated under hypoxia (Table S1). Stability and activation of HIF-1, the master regulator to adaptation to hypoxia, are regulated both at the level of oxygenation and by the length of hypoxic exposure [42]. HIF-1 not only regulates protein expression in response to hypoxia but also affects oxidoreductase activity (Table S2) and thereby its own degradation. In fact, HIF-1*α* has a very short half-life (*t*_1/2_ ~ 5 min) and is rapidly degraded in the presence of oxygen, often resulting in detection difficulties. It induces collagen-encoding genes such as prolyl (P4HA1, P4HA2, and P4HB) and lysyl (PLOD1 and PLODs) hydroxylases. P4HA1 and P4HA2 are required for deposition of collagen, whereas PLOD1 and PLOD2 form hydroxylysines in collagens, serving as glycosylation sites. Hydroxylysines are also essential for collagen cross-links and fiber alignment [43, 44]. In addition, a glycosyltransferase (GLT25D1) known to transfer *β*-galactose to hydroxylysine residues of collagen and the oxidoreductase ERO1-like protein alpha was found in close connection with these proteins in the STRING interaction network (Figure 2). Also these two proteins were found to be upregulated. Together, these HIF-induced proteins can mediate extracellular matrix (ECM) remodeling, thus promoting metastasis and invasion.

### 3.8. Cell Cycle Regulation

**Figure 4:**
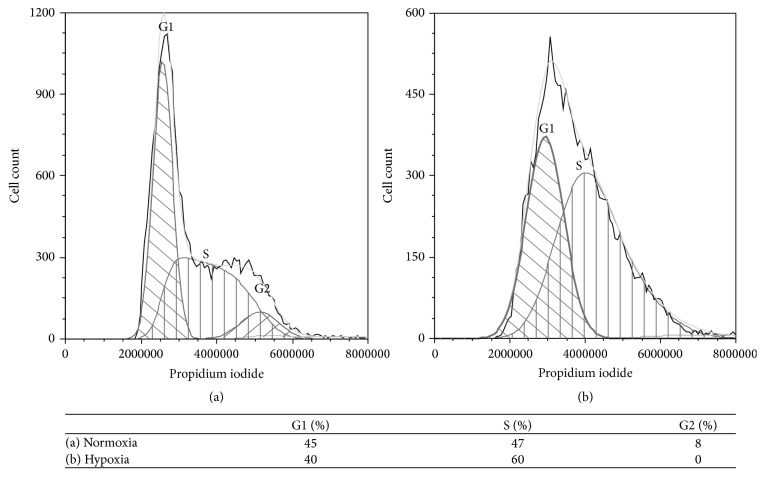
Cell cycle analysis. HeLa cells grown under (a) normoxic and (b) hypoxic conditions (1% O_2_), stained with propidium iodide. The premitotic phases G1, S, and G2 are represented in the figure (calculated from the fluorescence intensity values; grey line). Cells grown under hypoxic conditions differ from those exposed to normoxia by showing an accumulation in G1 and a prolonged S phase.

Cessation of growth is a common cellular response to hypoxia. HIF-1, important for adaptation to low levels of oxygen, may be a major regulator of cell cycle arrest during hypoxia [45]. Severe hypoxia (i.e., with pericellular oxygen concentrations well below 0.1%) specifically induces accumulation of cells in the S phase of the cell cycle [46]; however, hypoxia-induced damage of mammalian cells appears to depend on the cell cycle phase upon exposure to hypoxia. There are three distinct stages before mitosis, G1, S, and G2. Cells in G1 and G2 phase are considered more resistant to damage than those in S phase. At severe hypoxia, cells in G2 most likely proceed to the following G1 phase before arrest [47, 48]. By measuring the DNA content of individual cells, we obtained information about the cell cycle state. In HeLa cells grown under normoxic conditions, the G1, S, and G2 phases were distinctly observed in the DNA histogram. In our experiments under hypoxic conditions, the pericellular oxygen levels were established at 0.3%; hence no complete arrest occurred, but cells still showed an accumulation in G1 and a prolonged S phase (Figure 4). There are two oxygen-dependent checkpoints in early/mid or late G1, suggested to involve the proteins pRB and p27, respectively [47–49]. While p27 was not identified in our study, a pRB-associated protein was identified as downregulated (note that this protein did not fulfill our strict statistic criteria).

Some of the significantly upregulated proteins identified in the present study are involved in microtubule processes, among these the heavily upregulated N-myc downstream regulated protein (NDRG1) (Table S1). This protein regulates microtubule dynamics and protects cells from spindle disruption damage. Moreover, some DNA binding proteins, like DEAH (Table S1), important in chromatin binding, and the chromodomain-helicase-DNA-binding protein 3 (Table S1), responsible for chromatin organization, spindle organization, and centrosome integrity, were both downregulated in cells under hypoxic conditions. While little is known about the involvement of these proteins in hypoxia-induced cell cycle arrest, it has been suggested that they may protect cells from advancing further in the cell cycle [50]. Arguing that growth arrest prevents genomic instability, exposure of hypoxia to cancer cells may select for a more malignant phenotype.

## 4. Conclusion

Lack of oxygen is a hallmark of cancer and an important driving force to malignant progression, resulting in poor prognosis. Proteomic changes favoring survival under hypoxic conditions will lead to further enhanced hypoxia and more aggressive cell types. The response to hypoxia is complex; certainly, HIF transcription factors play an essential role in adaption to the hypoxic environment, but many other proteins and pathways are also involved in this response. By and large, the cell adapts to the limited levels of oxygen by adopting two major adaptation strategies: preserving energy by process slowdown and altering the metabolism to maximize energy gain under anaerobic conditions. In this work, we identified many proteins involved in the cellular response. As expected, we observed upregulation of all glycolytic enzymes as well as lactate dehydrogenase, while entry into the citric acid cycle was downregulated. More interestingly, we found 16 mitochondrial ribosomal proteins (MRPs) and six translocases of the outer and inner mitochondrial membrane (TOMM/TIMM) to be downregulated by hypoxia. This is to our knowledge the first time that mitochondrial clusters have been shown to be affected by hypoxia. Notably, the two mitochondrial protein clusters are connected to glycolysis by protein-protein interactions* via* downregulated C1QBP and PMPCB, respectively (Figure 2). The mitochondrial import machinery is important for the translocation of cytosolic proteins to keep mitochondria intact and functional. Among these are the mitochondrial ribosomal proteins responsible for translation of the 13 mitochondrial proteins, which are all components of the electron transport chain. In summary, these results not only significantly improve our understanding of the response to hypoxia, but also reveal proteins important for malignant progression, which may be targeted in potential future therapies.

## Supplementary Material

Supplementary Table 1: Proteins exhibiting altered regulation profiles in HeLa cells under hypoxic conditions, as identified by SILAC-based quantitative proteomic analyses.
Supplementary Table 2: Pathway and functional annotation cluster analysis.
Supplementary Figure 1: Workflow of a SILAC experiment.
Supplementary Figure 2: Lactate and glucose concentrations in cells cultivated under normoxic or hypoxic conditions.
Supplementary Figure 3: Pie charts of PANTHER biological processes and molecular functions.
Supplementary Figure 4: Western blot analysis of selected regulated proteins.

## References

[1] Vaupel P., Kallinowski F., Okunieff P. (1989). Blood flow, oxygen and nutrient supply, and metabolic microenvironment of human tumors: a review. *Cancer Research*.

[2] Lee Y. M., Jeong C.-H., Koo S.-Y. (2001). Determination of hypoxic region by hypoxia marker in developing mouse embryos in vivo: a possible signal for vessel development. *Developmental Dynamics*.

[3] Semenza G. L. (2000). Hypoxia, clonal selection, and the role of HIF-1 in tumor progression. *Critical Reviews in Biochemistry and Molecular Biology*.

[4] Dewhirst M. W., Cao Y., Moeller B. (2008). Cycling hypoxia and free radicals regulate angiogenesis and radiotherapy response. *Nature Reviews Cancer*.

[5] Brizel D. M., Scully S. P., Harrelson J. M. (1996). Tumor oxygenation predicts for the likelihood of distant metastases in human soft tissue sarcoma. *Cancer Research*.

[6] Reynolds T. Y., Rockwell S., Glazer P. M. (1996). Genetic instability induced by the tumor microenvironment. *Cancer Research*.

[7] Wenger R. H. (2002). Cellular adaptation to hypoxia: O_2_-sensing protein hydroxylases, hypoxia-inducible transcription factors, and O_2_-regulated gene expression. *The FASEB Journal*.

[8] Semenza G. L. (1998). Hypoxia-inducible factor 1: master regulator of O_2_ homeostasis. *Current Opinion in Genetics & Development*.

[9] Racker E. (1974). History of the Pasteur effect and its pathobiology. *Molecular and Cellular Biochemistry*.

[10] Warburg O. (1956). On respiratory impairment in cancer cells. *Science*.

[11] Warburg O., Wind F., Negelein E. (1927). The metabolism of tumors in the body. *The Journal of General Physiology*.

[12] Denko N. C. (2008). Hypoxia, HIF1 and glucose metabolism in the solid tumour. *Nature Reviews Cancer*.

[13] Gatenby R. A., Gillies R. J. (2004). Why do cancers have high aerobic glycolysis?. *Nature Reviews Cancer*.

[14] DeBerardinis R. J., Sayed N., Ditsworth D., Thompson C. B. (2008). Brick by brick: metabolism and tumor cell growth. *Current Opinion in Genetics and Development*.

[15] Ebbesen P., Pettersen E. O., Gorr T. A. (2009). Taking advantage of tumor cell adaptations to hypoxia for developing new tumor markers and treatment strategies. *Journal of Enzyme Inhibition and Medicinal Chemistry*.

[16] Huang X.-D., Wang Z.-F., Dai L.-M., Li Z.-Q. (2012). Microarray analysis of the hypoxia-induced gene expression profile in malignant C6 glioma cells. *Asian Pacific Journal of Cancer Prevention*.

[17] Chi J.-T., Wang Z., Nuyten D. S. A. (2006). Gene expression programs in response to hypoxia: cell type specificity and prognostic significance in human cancers. *PLoS Medicine*.

[18] Sørensen B. S., Horsman M. R., Vorum H., Honoré B., Overgaard J., Alsner J. (2009). Proteins upregulated by mild and severe hypoxia in squamous cell carcinomas in vitro identified by proteomics. *Radiotherapy and Oncology*.

[19] Vorum H., Østergaard M., Hensechke P., Enghild J. J., Riazati M., Rice G. E. (2004). Proteomic analysis of hyperoxia-induced responses in the human choriocarcinoma cell line JEG-3. *Proteomics*.

[20] Stockwin L. H., Blonder J., Bumke M. A. (2006). Proteomic analysis of plasma membrane from hypoxia-adapted malignant melanoma. *Journal of Proteome Research*.

[21] Ren Y., Hao P., Dutta B. (2013). Hypoxia modulates A431 cellular pathways association to tumor radioresistance and enhanced migration revealed by comprehensive proteomic and functional studies. *Molecular and Cellular Proteomics*.

[22] Djidja M.-C., Chang J., Hadjiprocopis A. (2014). Identification of hypoxia-regulated proteins using MALDI-mass spectrometry imaging combined with quantitative proteomics. *Journal of Proteome Research*.

[23] Ong S.-E., Blagoev B., Kratchmarova I. (2002). Stable isotope labeling by amino acids in cell culture, SILAC, as a simple and accurate approach to expression proteomics. *Molecular & Cellular Proteomics*.

[24] Ong S.-E., Foster L. J., Mann M. (2003). Mass spectrometric-based approaches in quantitative proteomics. *Methods*.

[25] Pettersen E. O., Larsen L. H., Ramsing N. B., Ebbesen P. (2005). Pericellular oxygen depletion during ordinary tissue culturing, measured with oxygen microsensors. *Cell Proliferation*.

[26] Cox J., Mann M. (2008). MaxQuant enables high peptide identification rates, individualized p.p.b.-range mass accuracies and proteome-wide protein quantification. *Nature Biotechnology*.

[27] Cox J., Neuhauser N., Michalski A., Scheltema R. A., Olsen J. V., Mann M. (2011). Andromeda: a peptide search engine integrated into the MaxQuant environment. *Journal of Proteome Research*.

[28] da Huang W., Sherman B. T., Lempicki R. A. (2009). Bioinformatics enrichment tools: paths toward the comprehensive functional analysis of large gene lists. *Nucleic Acids Research*.

[29] Huang D. W., Sherman B. T., Lempicki R. A. (2009). Systematic and integrative analysis of large gene lists using DAVID bioinformatics resources. *Nature Protocols*.

[30] Vizcaíno J. A., Deutsch E. W., Wang R. (2014). ProteomeXchange provides globally co-ordinated proteomics data submission and dissemination. *Nature Biotechnology*.

[31] Franceschini A., Szklarczyk D., Frankild S. (2013). STRING v9.1: protein-protein interaction networks, with increased coverage and integration. *Nucleic Acids Research*.

[32] Mi H., Muruganujan A., Thomas P. D. (2013). PANTHER in 2013: modeling the evolution of gene function, and other gene attributes, in the context of phylogenetic trees. *Nucleic Acids Research*.

[33] Harris A. L. (2002). Hypoxia—a key regulatory factor in tumour growth. *Nature Reviews Cancer*.

[34] O'Rourke J. F., Pugh C. W., Bartlett S. M., Ratcliffe P. J. (1996). Identification of hypoxically inducible mRNAs in HeLa cells using differential-display PCR—role of hypoxia-inducible factor-1. *European Journal of Biochemistry*.

[35] Semenza G. L., Roth P. H., Fang H.-M., Wang G. L. (1994). Transcriptional regulation of genes encoding glycolytic enzymes by hypoxia-inducible factor 1. *Journal of Biological Chemistry*.

[36] Choi S., Cho K., Kim J. (2009). Comparative proteome analysis using amine-reactive isobaric tagging reagents coupled with 2D LC/MS/MS in 3T3-L1 adipocytes following hypoxia or normoxia. *Biochemical and Biophysical Research Communications*.

[37] Halestrap A. P., Wilson M. C. (2012). The monocarboxylate transporter family-role and regulation. *IUBMB Life*.

[38] Anderson S., Bankier A. T., Barrell B. G. (1981). Sequence and organization of the human mitochondrial genome. *Nature*.

[39] Dolezal P., Likic V., Tachezy J., Lithgow T. (2006). Evolution of the molecular machines for protein import into mitochondria. *Science*.

[40] Neupert W., Herrmann J. M. (2007). Translocation of proteins into mitochondria. *Annual Review of Biochemistry*.

[41] Schmidt O., Pfanner N., Meisinger C. (2010). Mitochondrial protein import: from proteomics to functional mechanisms. *Nature Reviews Molecular Cell Biology*.

[42] Koumenis C., Wouters B. G. (2006). ‘Translating’ tumor hypoxia: unfolded protein response (UPR)-dependent and UPR-independent pathways. *Molecular Cancer Research*.

[43] Gilkes D. M., Bajpai S., Chaturvedi P., Wirtz D., Semenza G. L. (2013). Hypoxia-inducible factor 1 (HIF-1) promotes extracellular matrix remodeling under hypoxic conditions by inducing P4HA1, P4HA2, and PLOD2 expression in fibroblasts. *The Journal of Biological Chemistry*.

[44] Gilkes D. M., Bajpai S., Wong C. C. (2013). Procollagen lysyl hydroxylase 2 is essential for hypoxia-induced breast cancer metastasis. *Molecular Cancer Research*.

[45] Goda N., Ryan H. E., Khadivi B., McNulty W., Rickert R. C., Johnson R. S. (2003). Hypoxia-inducible factor 1*α* is essential for cell cycle arrest during hypoxia. *Molecular and Cellular Biology*.

[46] Giaccia A. J. (1996). Hypoxic stress proteins: survival of the fittest. *Seminars in Radiation Oncology*.

[47] Graff P., Åmellem Ø., Seim J., Stokke T., Pettersen E. O. (2005). The role of p27 in controlling the oxygen-dependent checkpoint of mammalian cells in late G1. *Anticancer Research*.

[48] Åmellem Ø., Sandvik J. A., Stokke T., Pettersen E. O. (1998). The retinoblastoma protein-associated cell cycle arrest in S-phase under moderate hypoxia is disrupted in cells expressing HPV18 E7 oncoprotein. *British Journal of Cancer*.

[49] Gardner L. B., Li Q., Park M. S., Flanagan W. M., Semenza G. L., Dang C. V. (2001). Hypoxia inhibits G_1_/S transition through regulation of p27 expression. *The Journal of Biological Chemistry*.

[50] Åmellem Ø., Pettersen E. O. (1991). Cell inactivation and cell cycle inhibition as induced by extreme hypoxia: the possible role of cell cycle arrest as a protection against hypoxia-induced lethal damage. *Cell Proliferation*.

