# Supplementary material for: Hypoxia Strongly Affects Mitochondrial Ribosomal Proteins and Translocases, as Shown by Quantitative Proteomics of HeLa Cells
Source: Int J Proteomics. 2015 Sep 2;2015:678527. doi: 10.1155/2015/678527 (PMC4572459; doi:10.1155/2015/678527)
Supplement: Supplementary file 1 — Supplementary Table 1: Proteins exhibiting altered regulation profiles in HeLa cells under hypoxic conditions, as identified by SILAC-based quantitative proteomic analyses. Supplementary Table 2: Pathway and functional annotation cluster analysis. Supplementary Figure 1: Workflow of a SILAC experiment. Supplementary Figure 2: Lactate and glucose concentrations in cells cultivated under normoxic or hypoxic conditions. Supplementary Figure 3: Pie charts of PANTHER biological processes and molecular functions. Supplementary Figure 4: Western blot analysis of selected regulated proteins. [file 678527.f1.pdf]

SUPPLEMENTARY INFORMATION

Hypoxia strongly affects mitochondrial ribosomal proteins and translocases, as shown by quantitative proteomics of HeLa cells

Paula A. Bousquet[1,5*], Joe Alexander Sandvik[2], Magnus Ø. Arntzen[3,5], Nina F. Jeppesen Edin[2], Stine Christoffersen[2], Ute Krengel[1], Erik O. Pettersen[2], Bernd Thiede[3,4]

[1]Department of Chemistry, University of Oslo, P.O 1033 Blindern, NO-0315 Oslo, Norway

[2]Department of Physics, University of Oslo, P.O 1048 Blindern, NO-0315 Oslo, Norway

[3]The Biotechnology Centre of Oslo, University of Oslo, P. O. Box 1125 Blindern, NO-0317 Oslo, Norway

[4] Department of Biosciences, University of Oslo, P. O. Box 1066 Blindern, NO-0316 Oslo, Norway

[5] Present addresses: Paula A. Bousquet, Department of Oncology, Akershus University Hospital, P.O. Box 1000, NO-1478 Lørenskog, Norway; Magus Ø. Arntzen, Department of Chemistry, Biotechnology and Food Science, Norwegian University of Life Sciences, P.O. Box 5003, NO-1432 Ås, Norway

***Corresponding author:** Paula A. Bousquet, Department of Chemistry, University of Oslo, P.O 1033 Blindern, NO-0315 Oslo, Norway.

Email: paula.bousquet@kjemi.uio.no

Supplementary Tables

Table S1. Proteins exhibiting altered regulation profiles in HeLa cells under hypoxic conditions, as identified by SILAC-based quantitative proteomic analyses (in alphabetical order). Three independent replicates were analyzed, and the normalized SILAC ratios of proteins and their corrected p-values are given (tested against the null hypothesis that there is no regulation). The variability was calculated from at least two log-transformed quantification values per protein.

Protein name	Gene ontology/ biological process	Uniprot accession (Gene name)	Ratio (H/L)	Corrected p-value	Variability
Down-regulated proteins					
3-hydroxyisobutyryl-CoA hydrolase, mitochondrial	lipid metabolism	Q6NVY1 (HIBCH)	0.50	0.009	0.057
Apolipoprotein B-100	recognition signal for the cellular binding and internalization of LDL	P04114 (APOB)	0.06	< 0.001	0.092
Aspartate aminotransferase, mitochondrial	amino acid metabolism	P00505 (GOT2)	0.58	0.064	0.063
Bifunctional methylenetetrahydrofolate dehydrogenase/cyclohydrolase, mitochondrial	NAD-dependent bifunctional enzyme	P13995 (MTHFD2)	0.47	0.004	0.067
Carbamoyl-phosphate synthase [ammonia], mitochondrial	amino acid metabolism, nitrogen metabolism	P31327 (CPS1)	0.59	0.069	0.082
Carnitine O-palmitoyltransferase 1, liver isoform	fatty acid metabolism	P50416 (CPT1A)	0.46	0.003	0.038
Collagen alpha-1(XII) chain	cell adhesion, extracellular structural organization	Q99715 (COL12A1)	0.32	< 0.001	0.102
Complement component 1 Q subcomponent-binding protein, mitochondrial	complement binding	Q07021 (C1QBP)	0.60	0.095	0.061
Cytochrome c oxidase assembly factor 6 homolog	cytochrome-c oxidase activity	Q5JTJ3 (C1orf31)	0.60	0.095	0.040
Dynamin-like 120 kDa protein, mitochondrial	microtubule-based process, intracellular transport	O60313 (OPA1)	0.56	0.046	0.063
Electron transfer flavoprotein subunit alpha, mitochondrial	generation of precursor metabolites and energy	P13804 (ETFA)	0.59	0.077	0.036
Ferritin, light polypeptide	ion binding, ion transport	P02792 (FTL)	0.55	0.035	0.103
Heat shock protein 75 kDa, mitochondrial	protein folding	Q12931 (TRAP1)	0.55	0.036	0.037
Hydroxysteroid dehydrogenase-like protein 2	oxidation reduction	Q6YN16 (HSDL2)	0.59	0.080	0.065
Isocitrate dehydrogenase [NAD] subunit alpha, mitochondrial	citric acid cycle (TCA cycle)	P50213 (IDH3A)	0.57	0.057	0.074
Leucine-rich PPR-motif containing protein, mitochondrial	microtubule-based process, intracellular transport	P42704 (LRPPRC)	0.58	0.057	0.178
Medium-chain specific acyl-CoA	fatty acid metabolism	Q5HYG7	0.57	0.049	0.046

dehydrogenase, mitochondrial		(ACADM)			
Mimitin, mitochondrial	NADH dehydrogenase activity	Q8N183 (NDUFAF2)	0.58	0.065	0.059
Mitochondrial import inner membrane translocase subunit TIMM16	protein transport	Q9Y3D7 (TIMM16)	0.60	0.095	0.049
Mitochondrial import inner membrane translocase subunit TIMM23	protein transport, protein localization	O14925 (TIMM23)	0.56	0.039	0.051
Mitochondrial import inner membrane translocase subunit TIMM44	protein transport, protein localization	Q53G69 (TIMM44)	0.60	0.084	0.027
Mitochondrial import inner membrane Translocase subunit TIMM50 homolog	protein transport, protein localization	Q3ZCQ8 (TIMM50)	0.46	0.003	0.066
Mitochondrial import receptor subunit TOMM22 homolog	protein transport, protein localization	Q9NS69 (TOMM22)	0.50	0.011	0.034
Mitochondrial import receptor subunit TOMM40 homolog	protein transport, protein localization	O96008 (TOMM40)	0.55	0.031	0.043
Mitochondrial ribosomal protein L1	RNA processing	Q9BYD6 (MRPL1)	0.60	0.095	0.086
Mitochondrial ribosomal protein L11	translation	Q9Y3B7 (MRPL11)	0.52	0.015	0.065
Mitochondrial ribosomal protein L13	translation	Q9BYD1 (MRPL13)	0.51	0.011	0.086
Mitochondrial ribosomal protein L17	translation	Q9NRX2 (MRPL17)	0.46	0.003	0.052
Mitochondrial ribosomal protein L21	translation	Q7Z2W9 (MRPL21)	0.52	0.015	0.026
Mitochondrial ribosomal protein L22	translation	Q9NWU5 (MRPL22)	0.48	0.005	0.008
Mitochondrial ribosomal protein L41	translation	Q8IXM3 (MRPL41)	0.50	0.008	0.040
Mitochondrial ribosomal protein L43	translation	Q8N983 (MRPL43)	0.51	0.011	0.035
Mitochondrial ribosomal protein L49	translation	Q13405 (MRPL49)	0.47	0.004	0.052
Mitochondrial ribosomal protein L50	unknown function	Q8N5N7 (MRPL50)	0.52	0.015	0.037
Mitochondrial ribosomal protein L9	translation	Q9BYD2 (MRPL9)	0.50	0.009	0.001
Mitochondrial ribosomal protein S23	unknown function	Q9Y3D9 (MRPS23)	0.54	0.029	0.024
Mitochondrial ribosomal protein S28	unknown function	Q53G62 (MRPS28)	0.52	0.014	0.062
Mitochondrial ribosomal protein S29	apoptosis	P51398 (DAP3)	0.49	0.008	0.073
Mitochondrial ribosomal protein S34	unknown function	P82930 (MRPS34)	0.58	0.059	0.054
Mitochondrial ribosomal protein S7	translation	Q9Y2R9 (MRPS7)	0.41	< 0.001	0.089
Mitochondrial-processing peptidase subunit beta	peptidase activity	O75439 (PMPCB)	0.48	0.005	0.023
Peptidyl-prolyl cis-trans isomerase F, mitochondrial	protein folding, mitochondrion organization	P30405 (PPIF)	0.50	0.009	0.003
Phosphatidylglycerophosphatase and	phosphatase activity	Q8WUK0	0.58	0.063	0.025

protein-tyrosine phosphatase 1		(PTPMT1)			
Polymerase delta-interacting protein 2	unknown function	Q9Y2S7 (POLDIP2)	0.55	0.036	0.064
Prostaglandin E synthase 2	oxidoreductase activity, fatty acid metabolic process	Q9H7Z7 (PTGES2)	0.54	0.029	0.058
Protein NOXP20	neuronal cell development	Q8IWE2 (FAM114A1)	0,33	< 0,001	0.016
Protein TBRG4	nucleotide binding, cell cycle process	Q969Z0 (TBRG4)	0,58	0,061	0.015
Putative ATP-dependent RNA helicase DHX30 (DEAH box protein)	nucleotide binding	Q7L2E3 (DHX30)	0.55	0.037	0.014
Pyruvate dehydrogenase E1 component subunit alpha, somatic form, mitochondria	glycolysis/gluconeogenesis, citric acid cycle (TCA cycle)	P08559 (PDHA1)	0.57	0.053	0.058
Sequestosome-1	intracellular signaling cascade, apoptosis	Q13501 (SQSTM1)	0.45	0.002	0.139
SRA stem-loop-interacting RNA-binding protein, mitochondrial	regulation of transcription	Q9GZT3 (SLIRP)	0.55	0.031	0.030
Succinate dehydrogenase assembly factor 2, mitochondrial	mitochondrial electron transport	Q9NX18 (SDHAF2)	0.50	0.010	0.023
Transmembrane protein 126A	unknown function	Q9H061 (TMEM126A)	0.54	0.024	0.075
Up-regulated during skeletal muscle growth protein 5	unknown function	Q96IX5 (USMG5)	0.60	0.094	0.049
Up-regulated proteins					
1,2-dihydroxy-3-keto-5-methylthiopentene dioxygenase	amino acid and methionine biosynthesis	Q9BV57 (ADI1)	1.67	0.034	0.023
1,4-alpha-glucan branching enzyme	carbohydrate transport and metabolism	Q04446 (GBE1)	2.40	< 0.001	0.015
5'-nucleotidase	nucleotide transport and metabolism	P21589 (NT5E)	3.47	< 0.001	0.063
6-phosphofructokinase type C	glycolysis/gluconeogenesis, pentose phosphate pathway	Q01813 (PFKP)	1.78	0.014	0.004
6-phosphofructokinase, liver type	glycolysis/gluconeogenesis, pentose phosphate pathway	P17858 (PFKL)	2.27	< 0.001	0.021
Adenylate kinase 4, mitochondrial	purine metabolism	P27144 (AK4)	3.15	< 0.001	0.075
Alpha-enolase 1	glycolysis/gluconeogenesis	P06733 (ENO1)	1.81	0.011	0.025
Ankyrin repeat and zinc finger domain containing 1	ion binding	Q9H8Y5 (ANKZF1)	3.19	< 0.001	0.014
Annexin A8	homeostasis, lipid binding, ion binding	P13928 (ANXA8L2)	1.76	0.015	0.045
Aspartyl-tRNA synthetase	aminoacyl tRNA biosynthesis	P14868 (DARS)	1.62	0.051	0.003
ATP-binding cassette, sub-family B member 6, mitochondrial	cellular homeostasis	Q9NP58 (ABCB6)	1.76	0.015	0.156
Autophagy-related protein 9A	cellular response to stress	Q3ZAQ6 (ATG9A)	1.93	0.004	0.077
Carbonic anhydrase IX	participate in pH regulation	Q16790 (CA9)	17.6	< 0.001	0.040

CD109 antigen	enzyme inhibitor activity	Q6YHK3 (CD109)	1.53	0.095	0.061
CD63 antigen	regulation of endocytosis	P08962 (CD63)	1.74	0.019	0.042
Cellular retinoic acid binding protein 2	retinoic acid binding	P29373 (CRABP2)	2.58	< 0.001	0.036
Chloride intracellular channel 3	ion transport	Q5SQ17 (CLIC3)	1.64	0.045	0.084
Collagen alpha-1 (V) chain	member of group I collagen	P20908 (COL5A1)	1.60	0.058	0.102
Collagen alpha-1(VII) chain	cell adhesion	Q02388 (COL7A1)	1.57	0.073	0.089
Collagen alpha-2(IV) chain	anti-angiogenic and anti-tumor cell activity	P08572 (COL4A2)	1.62	0.053	0.073
Dihydropyrimidinase-related protein 2	deydropyrimidinase activity	Q16555 (DPYSL2)	1.90	0.005	0.007
Dihydropyrimidine dehydrogenase	pyrimidine metabolism	Q12882 (DPYD)	6.69	< 0.001	0.030
Dynein heavy chain 6, axonemal	microtubule-based process	Q9C0G6 (DNAH6)	1.72	0.022	0.037
EH domain binding protein 1-like protein 1	actin binding	Q8N3D4 (EHBP1L1)	1.54	0.089	0.022
ERO1-like protein alpha	generation of precursor metabolites and energy	Q96HE7 (ERO1L)	3.13	< 0.001	0.035
Fibronectin	cell adhesion	P02751 (FN1)	4.84	< 0.001	0.030
Follistatin-like protein 4	ion binding	Q6MZW2 (FSTL4)	1.60	0.057	0.158
Fructose-bisphosphate aldolase A	glycolysis/gluconeogenesis, pentose phosphate pathway	P04075 (ALDOA)	2.00	0.002	0.013
Fructose-bisphosphate aldolase C	glycolysis/gluconeogenesis	P09972 (ALDOC)	2.78	< 0.001	0.107
Gamma-enolase	glycolysis/gluconeogenesis	P09104 (ENO2)	3.94	< 0.001	0.024
Glucose phosphate isomerase	glycolysis/gluconeogenesis, pentose phosphate pathway	P06744 (GPI)	2.46	< 0.001	0.012
Glycogen synthase 1	sucrose metabolism, insulin signaling pathway	P13807 (GYS1)	2.17	< 0.001	0.016
Hexokinase 2	glycolysis/gluconeogenesis, amino acid metabolism	P52789 (HK2)	3.27	< 0.001	0.031
Integrin alpha-2	response to hypoxia, regulation of actin cytoskeleton	P17301 (ITGA2)	2.09	0.001	0.161
Integrin alpha-5	regulation of actin cytoskeleton, cell adhesion	P08648 (ITGA5)	3.05	< 0.001	0.032
Isocitrate dehydrogenase 2 (NADP), mitochondrial	citric acid cycle (TCA cycle)	P48735 (IDH2)	1.67	0.034	0.022
Lactate dehydrogenase A	glycolysis/gluconeogenesis, pyruvate metabolism	P00338 (LDHA)	2.92	< 0.001	0.009
Leucin-rich repeat-containing protein 8A	involved in B-cell development	Q8IWT6 (LRRC8A)	1.75	0.018	0.178
Lysophosphatidylcholine acyltransferase	phospholipid metabolic	Q8NF37	2.49	< 0.001	0.033

1	process	(LPCAT1)			
Macrophage migration inhibitory factor	amino acid metabolism	P14174 (MIF)	1.93	0.004	0.017
Monocarboxylate transporter, MCT4/5 (solute carrier family 16, member 3)	glucose transporter	Q53G91 (SLC16A3)	1.97	0.003	0.038
Peptidylprolyl isomerase C	protein folding	P45877 (PPIC)	1.68	0.031	0.023
Phosphoglucomutase-1	glycolysis/gluconeogenesis, pentose phosphate pathway	P36871 (PGM1)	2.05	0.001	0.003
Phosphoglycerate kinase 1	glycolysis/gluconeogenesis	P00558 (PGK1)	3.48	< 0.001	0.034
Phosphoglycerate kinase 2	glycolysis/gluconeogenesis	P07205 (PGK2)	3.24	< 0.001	0.010
Phosphoglycerate mutase 1	glycolysis/gluconeogenesis	P18669 (PGAM1)	1.87	0.006	0.009
Plexin-D1	morphogenesis of a branching structure	Q9Y4D7 (PLXND1)	1.90	0.005	0.027
Procollagen galactosyltransferase 1	carbohydrate and lipid biosynthetic process	Q8NBJ5 (GLT25D1)	1.63	0.049	0.016
Procollagen-lysine, 2-oxoglutarate 5-dioxygenase 1	response to hypoxia, lysine degradation, oxidoreductase activity	Q5JXB8 (PLOD1)	2.80	< 0.001	0.040
Procollagen-lysine, 2-oxoglutarate 5-dioxygenase 2	response to hypoxia, lysine degradation, oxidoreductase activity	Q59ED2 (PLOD2)	6.39	< 0.001	0.034
Prolyl 4-hydroxylase subunit alpha-1	amino acid metabolism, oxidoreductase activity	P13674 (P4HA1)	5.30	< 0.001	0.051
Prolyl 4-hydroxylase subunit beta	oxidoreductase activity	P07237 (P4HB)	1.80	0.011	0.021
Prolyl 4-hydroxylase, subunit alpha-2	amino acid metabolism, oxidoreductase activity	O15460 (P4HA2)	3.41	< 0.001	0.028
Protein FAM162A	may be involved in hypoxia-induced cell death	Q96A26 (FAM162A)	2.53	< 0.001	0.020
Protein NDRG1	cell activation	Q92597 (NDRG1)	11.51	< 0.001	0.040
Protein NOXP20	unknown function	Q8IWE2 (FAM114A1)	1.58	0.068	0.016
Protein S100-A10	calcium ion binding	P60903 (S100A10)	2.23	< 0.001	0.017
Protein S100-A16	response to metal ion	Q96FQ6 (S100A16)	1.62	0.051	0.087
Pyruvate kinase PKM	glycolysis/gluconeogenesis, pyruvate metabolism	P14618 (PKM2)	1.79	0.012	0.031
Receptor-type tyrosine-protein phosphatase F	cell adhesion, insulin signaling pathway, phosphatase activity	P10586 (PTPRF)	1.54	0.091	0.020
RelA-associated protein inhibitor	regulation of apoptosis	Q8WUF5 (PPP1R13L)	1.59	0.061	0.016
SH3 domain binding glutamic acid-rich-like protein 3	cell redox homeostasis	Q9H299 (SH3BGRL3)	1.79	0.013	0.050
Solute carrier family 2 (facilitated glucose transporter), member 1	glucose transporter	Q59GX2 (SLC2A1)	2.76	< 0.001	0.025
Solute carrier family 2 (facilitated glucose	glucose transporter	P11169	5.08	< 0.001	0.021

transporter), member 3		(SLC2A3)			
Solute carrier family 38, member 2	ion transport	Q96QD8 (SLC38A2)	1.94	0.004	0.024
Spermine synthase	carbohydrate transport and metabolism	P52788 (SMS)	1.55	0.082	0.008
Succinate dehydrogenase [ubiquinone] iron-sulfur subunit, mitochondrial	citric acid cycle (TCA cycle), oxidative phosphorylation	P21912 (SDHB)	1.88	0.006	0.042
Supervillin	cytoskeleton organization	Q569J5 (SVIL)	1.68	0.030	0.003
Thymidine kinase, cytosolic	translation	P04183 (TK1)	1.67	0.033	0.012
Transforming growth factor-beta-induced protein ig-h3	cell adhesion, extracellular structural organization	Q15582 (TGFBI)	2.42	< 0.001	0.036
Triosephosphate isomerase	glycolysis/gluconeogenesis	Q53HE2 (TPI1)	2.89	< 0.001	0.007
Tubulin alpha-1A chain	microtubule-based process	Q71U36 (TUBA1A)	1.91	0.004	0.017
Vimentin	cell motion	P08670 (VIM)	1.54	0.093	0.018

Table S2. Pathway and functional annotation cluster analysis. (A) KEGG pathway and (B) Gene Ontology based analysis of regulated proteins in HeLa cells in hypoxic environments. Enrichment scores are used to rank the biological significance. GO, gene ontology.

A

GO-Term	Enrichment score	Count	Regulation
Glycolysis/ Gluconeogenesis	8.05	14	Up
Pentose phosphate pathway	8.05	6	Up
Fructose and mannose metabolism	8.05	6	Up
Starch and sucrose metabolism	3.19	5	Up
Galactose metabolism	2.70	4	Up

B

GO-Term	Enrichment score	Count	Regulation
Glycolysis	8.05	14	Up
Mitochondrial part	6.62	30	Down
Ion binding	3.31	31	Up
Oxidoreductase activity	2.06	4	Up
Response to hypoxia	2.00	5	Up

Supplementary Figures

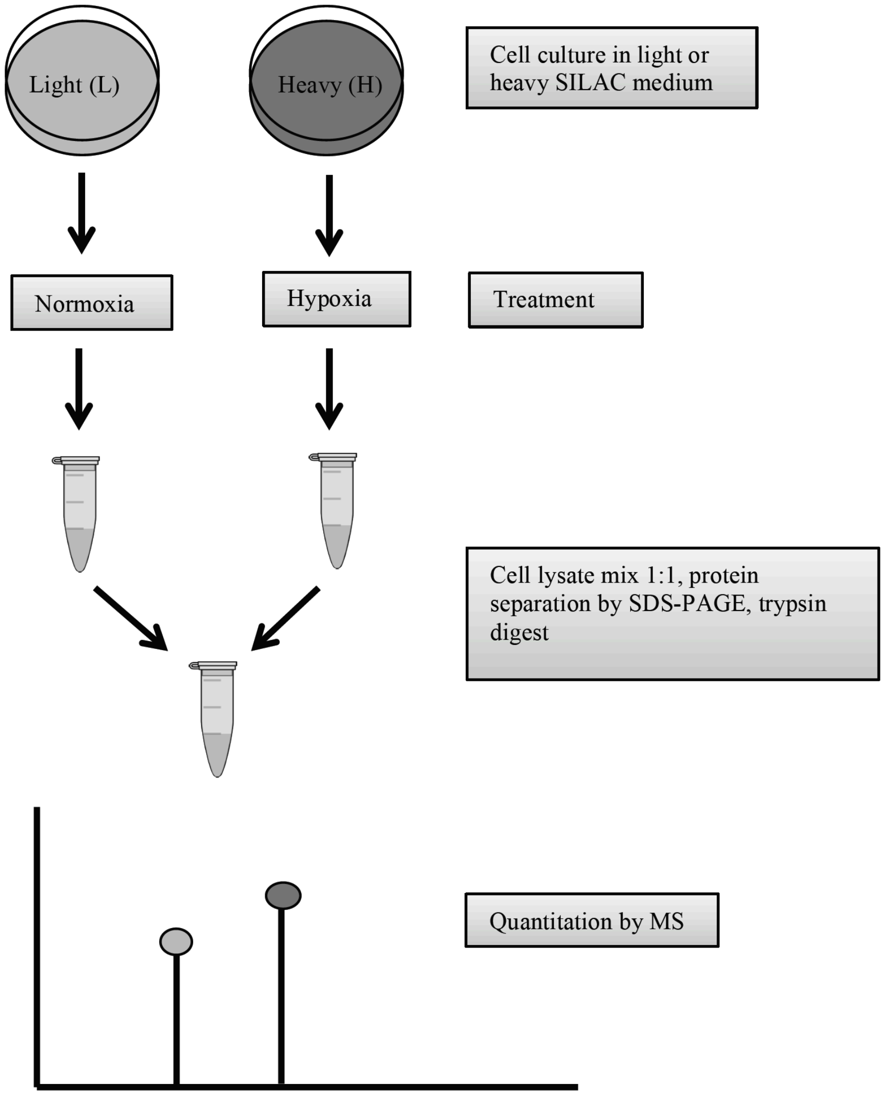

Figure S1. Workflow of a SILAC experiment. Two cell populations are labeled with either light or heavy amino acids, then combined and analyzed by LC-MS/MS. The differences in intensity in the signals between the light and heavy peptides are used to calculate the ratios between the samples.

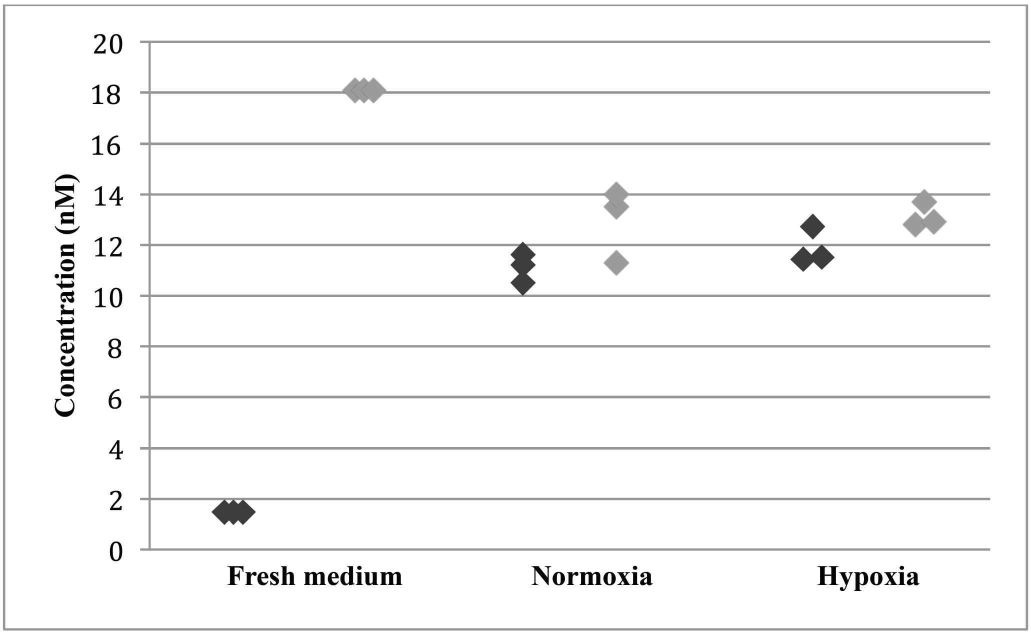

Figure S2. Lactate (dark grey) and glucose (light grey) concentrations in cells cultivated under normoxic or hypoxic conditions.

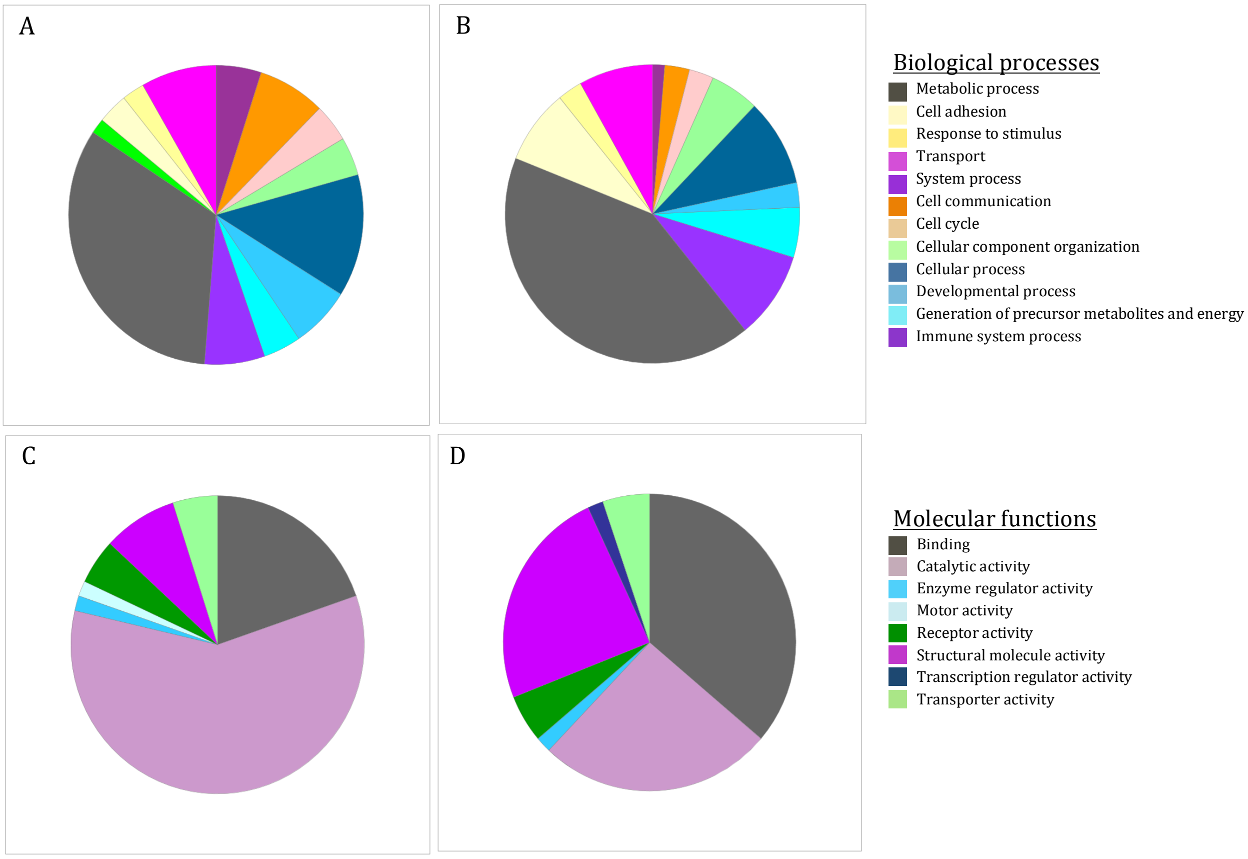

Figure S3. Pie charts of PANTHER biological processes and molecular functions. (A, B) Biological processes and (C, D) Molecular functions. Panels A and C refer to up-regulated proteins, while panels B and D concern down-regulated proteins.

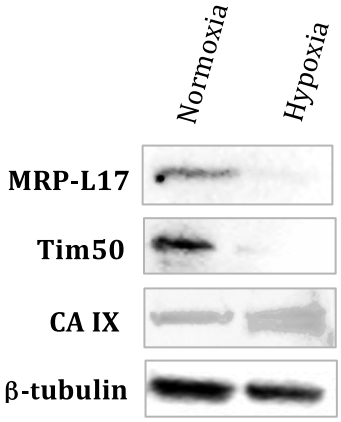

Figure S4. Representative Western blot analysis. Selected proteins are MRP-L17, Tim50 and CAIX. Shown are experiments from HeLa cells grown under normoxic (left lane) or hypoxic conditions (right lane). For comparison, the proteomics analysis yielded down-regulation for MRLP-L17 and Tim50 (H/L 0.46, $p=0.003$ for both proteins) and up-regulation for CA IX (H/L 17.6, $p<0.001$). The two bands for CA IX correspond to different CA IX isoforms. β-tubulin was used as a control, to demonstrate equal loading of the gels.

Supplementary Material and Methods

Oxygen sensing

Pericellular oxygen concentration and cellular respiration/consumption were performed as described [1]. Briefly, an oxygen sensor (Unisense, Denmark) fixed to a motor driver was mounted on an inverted microscope (Nikon TS100, Japan) with a Paxcam3 camera (MIS, IL, USA). The sensor (25 μm in diameter) was positioned through the open flask neck, allowing the measurement of the oxygen level inside the culture flask at the same time as image capturing (after the end point of the sensor tip was carefully positioned at the flask bottom close to the attached cells). Measurement of the oxygen concentration started above the surface of medium, and proceeded in steps of 100 μm through the medium until the bottom of the flask was reached. This procedure was automatized using the integrated measuring and motor control software Sensor Trace Profiler (Unisense, Denmark) and repeated every 2 hours during the experiment. Readings from the last point in each profile gave the real pericellular oxygen concentration. The oxygen concentration gradient along the depth of medium was used to calculate oxygen flux in real time. Combined with cell density data obtained from photographs taken at the same time points, the consumption of oxygen per cell was calculated. The microscope and oxygen measuring system were placed inside an InVivo2 400 hypoxia incubator box (Ruskinn Technology, UK) and pictures were taken every 24 hours. The oxygen level inside the box was set to 1% for hypoxia and 19% for normoxic conditions.

NanoLC-LTQ Orbitrap mass spectrometry

The cell lysates from each labeling, heavy and light, were mixed 1:1 and subjected to SDS-PAGE. Each Coomassie G-250 stained SDS-PAGE gel lane was cut into 12 slices, which were in-gel digested using 0.1 μg of trypsin in 25 μl of 50 mM ammonium bicarbonate, pH 7.8. After micropurification using μ-C18 ZipTips (Millipore, Oslo, Norway), the peptides were dried in a SpeedVac and dissolved in 10 μl 1% formic acid, 5% acetonitrile in water. Half of the volume was injected into

an Ultimate 3000 nanoLC system (Dionex, Sunnyvale CA, USA) connected to a linear quadrupole ion trap-orbitrap (LTQ-Orbitrap XL) mass spectrometer (ThermoScientific, Bremen, Germany) equipped with a nanoelectrospray ion source. For liquid chromatography separation, an Acclaim PepMap 100 column (C18, 3 µm beads, 100 Å, 75 µm inner diameter) (Dionex, Sunnyvale CA, USA) capillary of 50 cm bed length was used. The flow rate was 0.3 µL/ min, with a solvent gradient of 7% B to 35% B in 110 minutes. Solvent A was aqueous 0.1% formic acid, whereas solvent B was aqueous 90% acetonitrile in 0.1% formic acid. The mass spectrometer was operated in the data-dependent mode to automatically switch between Orbitrap-MS and LTQ-MS/MS acquisition. Survey full scan MS spectra (from m/z 300 to 2,000) were acquired in the Orbitrap with the resolution R = 60,000 at m/z 400. The method used allowed the sequential isolation of up to the seven most intense ions for fragmentation on the linear ion trap using collision induced dissociation (CID) at a target value of 10,000 charges. Target ions already selected for MS/MS were dynamically excluded for 60 seconds. The lock mass option was enabled in MS mode for internal recalibration during the analysis. Other instrument parameters were set as previously described [2].

Protein identification and quantification

Trypsin was selected for cleavage. For the analysis, two missed cleavage sites were allowed, and tolerance levels for identification were set to 10 ppm and 0.5 Da for MS and fragment MS/MS scans, respectively. In addition to ^{13}C isotopes of arginine and lysine, methionine oxidation, deamidation of asparagines and glutamines, N-terminal protein acetylation and conversion of N-terminal peptide glutamine to pyro-glutamic acid were selected as variable modifications.

Supplementary References

1. Pettersen EO, Larsen LH, Ramsing NB, Ebbesen P. Pericellular oxygen depletion during ordinary tissue culturing, measured with oxygen microsensors. Cell Prolif. 2005;38(4):257-67. Epub 2005/08/16.

2. Koehler CJ, Strozynski M, Kozielski F, Treumann A, Thiede B. Isobaric peptide termini labeling for MS/MS-based quantitative proteomics. J Proteome Res. 2009;8(9):4333-41. Epub 2009/08/07.
